# Influenza virus antigenic variation, host antibody production and new approach to control epidemics

**DOI:** 10.1186/1743-422X-6-30

**Published:** 2009-03-13

**Authors:** Jiezhong Chen, Yi-Mo Deng

**Affiliations:** 1 John Curtin School of Medical Research, Australian National University, Canberra, ACT 2601, Australia; 2 WHO Collaborating Centre for Reference and Research on Influenza, North Melbourne, VIC 3051, Australia

## Abstract

Influenza is an infectious disease and can lead to life-threatening complications like pneumonia. The disease is caused by three types of RNA viruses called influenza types A, B and C, each consisting of eight negative single-stranded RNA-segments encoding 11 proteins. Current annual vaccines contain two type A strains and one type B strain and are capable of inducing strong antibody responses to both the surface glycoprotein hemagglutinin and the neuraminidase. While these vaccines are protective against vaccine viruses they are not effective against newly emerging viruses that contain antigenic variations known as antigenic drift and shift. In nature, environmental selection pressure generally plays a key role in selecting antigenic changes in the antigen determining spots of hemagglutinin, resulting in changes in the antigenicity of the virus. Recently, a new technology has been developed where influenza-specific IgG^+ ^antibody-secreting plasma cells can be isolated and cloned directly from vaccinated humans and high affinity monoclonal antibodies can be produced within several weeks after vaccination. The new technology holds great promise for the development of effective passive antibody therapy to limit the spread of influenza viruses in a timely manner.

## Background

Influenza is an infectious disease with symptoms of the common cold such as chills, high fever, sore throat, muscle pains, severe headache, coughing, bleeding from nose, weakness and general discomfort, but it is a much more severe disease as it can lead to life-threatening complications (like pneumonia) and death. Influenza is caused by three types of RNA viruses called influenza types A, B and C, which all belong to the orthomyxoviridae family. The so called "flu" in humans is generally caused by the viruses A and B, which are transmitted by aerosols from infected individuals or through contact with infected animals [1]. The disease mainly attacks weaker populations like children, old people and immune incompetent patients. Historically, flu epidemics are responsible for the deaths of millions of people. At present there is fear of pandemics of aggressive avian H5N1, which has already caused 382 cases of infection and 241 deaths according to WHO statistics [2-4]. Structurally, each influenza virus consists of eight negative single-stranded RNA-segments encoding 11 proteins [2]. The current vaccine regime against influenza is protective, which usually includes 2 strains of type A and 1 strain of type B capable of producing strong antibody responses to the surface glycoprotein hemagglutinin (HA) and neuraminidase (NA) of these viruses. However, like other RNA viruses, the HA and NA antigens are highly variable, and this makes it difficult to control new epidemics of influenza. Recent years have seen significant progress in this field, as exemplified by the following two recent studies. The first study details the relationship between changing environmental selective pressure and antigenic changes in human influenza [5], and the second one reports the identification of time-dependent antibody response to an influenza vaccine virus and rapid production of high affinity, virus-specific human monoclonal antibodies [6]. These progresses will prove to be essential for the development of effective medical countermeasures to cope with influenza infection and epidemics.

## Antigen evolution pattern

Influenza antigenic properties are determined by both HA and NA [7]. HA acts to attach the virus into host cells and subsequently fuse it to cell membranes, which is essential for the virus life cycle [8]. HA is synthesised as a single peptide but cleaved into HA1 and HA2 by specific host protease. The amino acids at the cleavage site are important in determining the virulence of the virus, that is the virus becomes highly virulent if these amino acids are lipophilic, [8]. Immunity induced by HA has been shown to increase host resistance to influenza and reduce the likelihood of infection and severity [9]. However, such protection is not effective against newly emerging influenza viruses that contain antigenic variations known as antigenic drift and shift [10]. Antigenic drift refers to a minor change (such as amino acid substitution in HA and/or NA) resulting in antigenic site change. In contrast, antigenic shift is the formation of a new virus subtype with mixed HA and NA from different subtypes. How do these alterations occur? It has been shown that selection pressure in the environment plays a key role in selecting antigenic changes in the antigen determining spots of HA, such as in places undergoing adaptive evolution and in antigenic locations undergoing substitutions, resulting in changes in the antigenicity of the virus [5]. It is also known that glycosylation of HA does not correlate with either the antigenicity or the selection pressure [5]. This process represents the side of the pathogen to escape the host defence through co-evolution with the host.

## Antibody response to influenza infection

In the host, infection by an influenza virus triggers a series of immune responses to counteract the invading virus. Antibody response has been shown to play an important role in protection against influenza virus infection [11]. Recently, Wrammert and colleagues demonstrated that IgG^+ ^antibody-secreting plasma cells (ASCs) increase rapidly to the highest level at day 7 after vaccination and then return to minimal levels at day 14 while influenza-specific memory B-cells peaks at day14–21 [6]. These ASCs are newly divided rather than pre-existing as demonstrated by the expression of the human leukocyte antigen and the proliferation of antibody marker Ki-67 [6]. They also demonstrated that the original antigen sin (OAS), which refers to higher affinity for a previously encountered virus than for the virus strain present in the vaccine, is unlikely in the normal and healthy adults [6]. Most of the secreted antibodies are specific to the vaccine virus. Among 86 monoclonal antibodies isolated, 61 have high binding affinity for the vaccine virus [6], the majority (60%) of which are against HA with the rest against NA, nucleoprotein (NP) and other antigens. The antibodies are also shown to be able to neutralize viral infection in MDCK cells, and therefore, they could play a crucial role in limiting the spread of the virus [6].

## Rapid antibody production and therapeutic implications

The current drug therapy for influenza infection is not satisfactory. At present, only two classes of drugs have been licensed for human use: M2 ion channel inhibitors (amantadine, rimantadine) and neuraminidase inhibitors (oseltamivir, zanamivir, peramivir). However, clinically oseltamivir phosphate does not bring survival rate up [12-14]. Strains that are resistant to amantadine have also emerged which may compromise the current drug therapy [15,16]. Undoubtly, the recent finding by Wrammert et al [6] holds great promise for the development of passive antibody therapy against the spread of influenza viruses because (1) high affinity human monoclonal antibodies can be produced in less than a month after vaccination and (2) these antibodies, because of their human origin, will have no or minimal antibody-related side-effects in humans.

## Role of intact host immune responses

Host responses against influenza include an intact and functional cascade of changes including both innate and adaptive immunities such as cytokine and interferon production, macrophage function, dendritic cells, natural killer cell function; and cytotoxic T lymphocyte activity, influenza-specific antibodies. In mice, defects in interferon α and β, complement system have been shown to increase morbidity and mortality [17,18]. Natural killer cells and macrophages are very early responses which are critical for the host to counteract the infection [19]. CD8+ T cells clear influenza virus by perforin-mediated pathways [20,21]. Mice deficient in CD8+ T-cells have increased viral replication and morbidity after infected with PR8 [22]. CD4+ T-cells are essential for effective B-cell responses to produce antibodies [21]. Plasmacytoid dendritic cells (pDCs) can internalise viral antigens and present them on major histocompatibility complex (MHC) class I to CD8+ T-cells to enhance host immune responses [23]. These defence mechanisms when work together are very effective against virus infection. However, there are also cases where the stimulation of only some of the elements in the immune system may be required to synergize the use of anti-influenza antibodies. Potentially, anti-M2 antibody may also be used against influenza infection as M2 is not variable compared to HA and NA [24].

In summary, the clinical outcome of influenza depends on both the influenza virus and the host defence. Recent studies have furthered our understanding about virus antigenic variation to escape the host immune system. Recent advances, particularly, in rapid production of human antibodies to influenza viruses will help develop new medical countermeasures to control influenza epidemics.

## Competing interests

The authors declare that they have no competing interests.

## Authors' contributions

JC and YD jointly wrote the manuscript, read and approved it.
